# Supplementary material for: Oncogenic KRAS-Induced Protein Signature in the Tumor Secretome Identifies Laminin-C2 and Pentraxin-3 as Useful Biomarkers for the Early Diagnosis of Pancreatic Cancer
Source: Cancers (Basel). 2022 May 27;14(11):2653. doi: 10.3390/cancers14112653 (PMC9179463; doi:10.3390/cancers14112653)
Supplement: Supplementary file 1 [file cancers-14-02653-s001.zip › File S1.pdf]

APPENDIX A - SUPPLEMENTAL DATA

Supplemental Material and Methods

Proteomics analysis by SILAC-MS

Stable Isotope Labeling by Amino Acids in Cell Culture (SILAC). SILAC media were prepared as follow. Light (L) and heavy (H) SILAC media were prepared by supplementing FBS RPMI 1640 (Lonza, BioWhittaker) with 0.47 mM L-arginine-HCl plus 0.46 mM L-Lysine-2HCl or 0.47 mM 13C615N4 L-arginine-HCl plus 0.46 mM 13C6 L-lysine-2HCl, respectively (Pierce Thermo Scientific). Media were filtered through a 0.22 μm filter (ALBET).

Cells lines were cultured in duplicate as described under paragraph 2.1. Once the cells had reached 80-90% confluence, the medium was discarded and replaced with SILAC medium. After six cell doublings, when cells reached 90% confluence, the medium was discarded and flasks were rinsed 7 times with PBS to minimize the amount of contaminating FBS proteins. Cells were then incubated with FBS-free SILAC medium for 24 hours at 37°C. The conditioned media (CM) from the duplicate flasks (each containing about 2.5x107 cells in 35 mL CM) were collected, pooled, added with protease inhibitor mixture (complete EDTA-free protease inhibitor cocktail tablets, Roche), and filtered through a 0.22 μm filter (AL-BET). After collecting the CM, cell viability was measured by Trypan blue test.

Global quantitative proteomic analysis by SILAC mass-spectrometry (MS). Each of the six clones was directly compared –by co-analysis– with the same Mock control cell line. In three separate experiments, the following three sample pairs were analyzed in parallel: Mock(H)/Mock(L), E-Clone(H)/Mock(L), and M-Clone(H)/Mock(L). To prepare each H/L SILAC sample pair, (H)- and (L)-CM were mixed 1:1 by protein content, and concentrated using 3 kDa MWCO Amicon Ultra centrifugal filter devices (Millipore) (5000xg, 4°C). Each retentate was then buffer-exchanged with water, and concentrated to 30 μL on a 3 kDa MWCO Ultrafree-0.5 Centrifugal Filter Unit (Millipore) (12000xg, 4°C). For each sample pair, 30 μg of proteins were separated by 1DE, and the gel lane obtained was cut into 24 slices of equal height. Proteins were in-gel digested, as described by Schiarea et al. [1].

An aliquot of each digest (2 μL) was analyzed by LC-MS/MS in the data-dependent mode, using a LTQ Orbitrap XL™ interfaced with an Agilent 1200 series capillary pump, as described by Schiarea et al. [1]. Each digest (2 μL) was then re-analyzed using the "precursor ion exclusion" method (i.e. with the guided exclusion –from the data-dependent MS/MS run– of the ions already selected in the first run). The 48 raw data files obtained from the LC-MS/MS analysis of each gel lane (i.e. MS/MS data for all proteins in a sample), were processed together in a multi-file project by Mascot Distiller software (version 2.4.3.3, Matrix Science) for protein identification and quantitation. The Mascot Distiller peak list of each sample was submitted to an in-house Mascot server 2.4.0 for searching against both the SwissProt v56.5 (537,505 sequences; 190,795,142 residues) and contaminants database 20100513 (248 sequences; 128,465 residues). Mascot search parameters were: taxonomy, Homo sapiens (20,250 sequences); enzyme, Trypsin with one missed cleavage allowed; fixed modification, cysteine carbamidomethylation; variable modifications, methionine oxidation; mass tolerance for peptide precursor ions 5 ppm; mass tolerance for MS/MS fragment ions, 0.8 Da; peptide charge, 2+, 3+, 4+. The significance threshold was set at $p<0.01$ and the cut-off for peptide ion score was set to >30, a value

ensuring an identification confidence >99.9%. SILAC quantitation was performed by Mascot Distiller using "SILAC K+6 R+10" quantitation method. To be accepted, peptides needed to pass the following quality thresholds: correlation, 0.95; fraction, 0.3; standard error, 0.2. Each protein quantification value was determined by Mascot Distiller as the median of the quantification values of its constituent peptides. Only the proteins identified and quantified by at least two non-redundant peptides were accepted as valid. We eliminated from the list all the proteins identified as "contaminant", like keratins and FBS proteins. To correct ex post any experimental imprecision in the mixing 1:1 proteins of each H/L sample pairs, all H/L ratios in a sample were normalized by dividing them by the actual median H/L value, so as to center the experimental median to the theoretical value of 1. Normalized H/L ratios were then expressed as log2(H/L).

In order to generate a list of proteins with changed secretion levels in the cloned cell lines compared to Mock, we established a cut-off value corresponding to 3SD of the normal distribution of log2 ratio (H/L) of all proteins quantified in the three control pairs (MH/ML), i.e. ±0.8916.

Supplemental Results

Secretome stability. Overall secretome stability in the control sample (Mock). Keeping in mind that in a SILAC experiment a log2(H/L)=0 indicates perfect stability (i.e. an identical protein amount in the H and L components of a "sample relative to control" or H/L pair), the 1119 redundant proteins quantified in our three MH/ML replicate samples showed rather stable levels (log2 H/L value distribution: mean±SD=0.013±0.297, n=1119). Within this Mock-H/Mock-L dataset, we found 11 proteins with a log2 H/L value exceeding ±0.8916, i.e. ±3SD, which represents our dysregulation cut-off. These 11 proteins were therefore excluded from our final dataset of 600 proteins. These quantitative results proved that – within three biological replicates of control H/L Mock pairs– the variability was low, and thus the use of "Mock-Light secretomes" as internal controls for our "KrasG12V-Heavy secretomes" was appropriate.

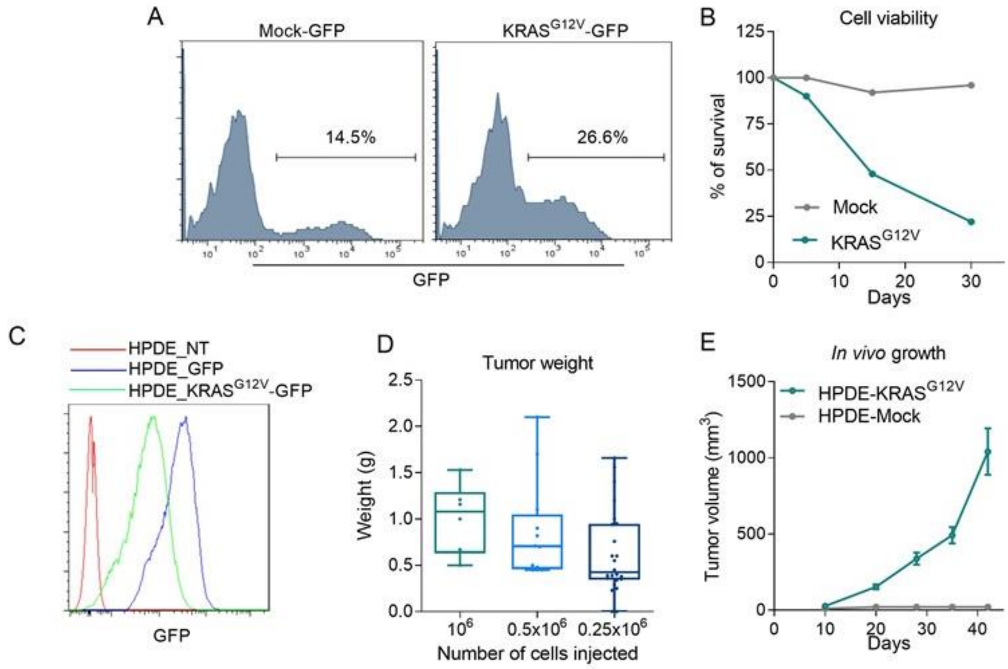

Figure S1. Transduction of the oncogenic *KRAS^G12V^* in human pancreatic ductal cells.

A) Flow cytometry analysis of primary cultures of ductal epithelial cells from normal human pancreatic samples (adjacent to pathological pancreatic tissues undergoing surgery) 7 days after infection with the GFP-lentiviral vector (pRRL.sinPPT.CMV.GFPpre-K-RAS^{G12V}) carrying the cDNA of the oncogenic *KRAS*^{G12V}.

B) In vitro cell viability over time of primary human epithelial pancreatic cells transduced with the oncogenic *KRAS*^{G12V} or mock-transduced.

C) Flow cytometry analysis of HPDE cells transduced with the *KRAS*^{G12V} GFP-lentiviral vector.

D) In vivo growth ability of HPDE- *KRAS*^{G12V} cells injected in SCID mice at different cell concentrations. Tumor weight after 30 days. The Mock-transduced HPDE cells were unable to generate tumors.

E) In vivo tumor growth curve of a representative tumor generated after injection of HPDE-KRAS^{G12V} cells in SCID mice.

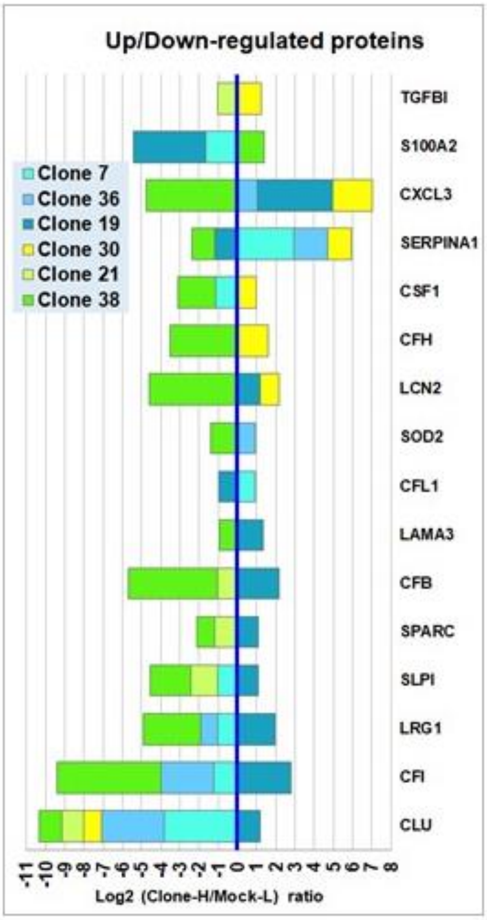

Figure S2. Divergent dysregulations of 16 proteins in the KRAS-cloned lines.

Distribution of multiple diverging dysregulations of secreted proteins across the 6 KRAS-clones (H), each paired with a Mock (L) sample, ob-tained by SILAC-MS quantitative proteomics (dysregulated values only).

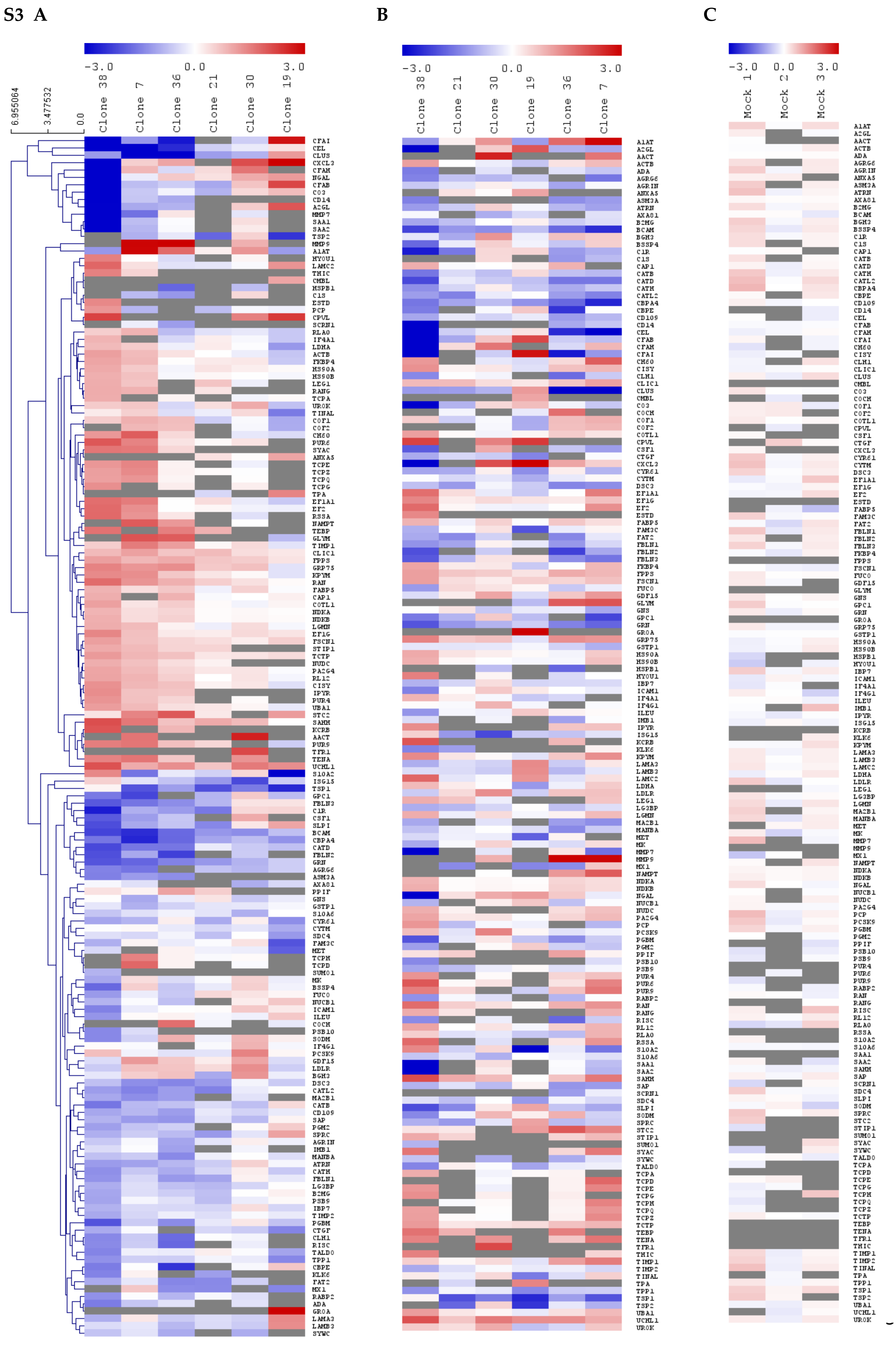
S3 A
-3.0 0.0 3.0
6.955064
3.477532
0.0
Clone 38
Clone 7
Clone 36
Clone 21
Clone 30
Clone 19
B
-3.0 0.0 3.0
Clone 38
Clone 21
Clone 30
Clone 19
Clone 36
Clone 7
C
-3.0 0.0 3.0
Mock 1
Mock 2
Mock 3

Figure S3 – Heatmaps of the 163 dysregulated proteins in the secretome profiles of six KRAS-cloned cell lines and three Mock samples.

A) heatmap based on Euclidean distance hierarchical clustering of the 163 dysregulated proteins identified in the six KRAS-cloned cell lines. B-C heatmaps of the 163 dysregulated proteins (in alphabetical order) for B) KRAS-cloned cell lines and C) Mock samples.

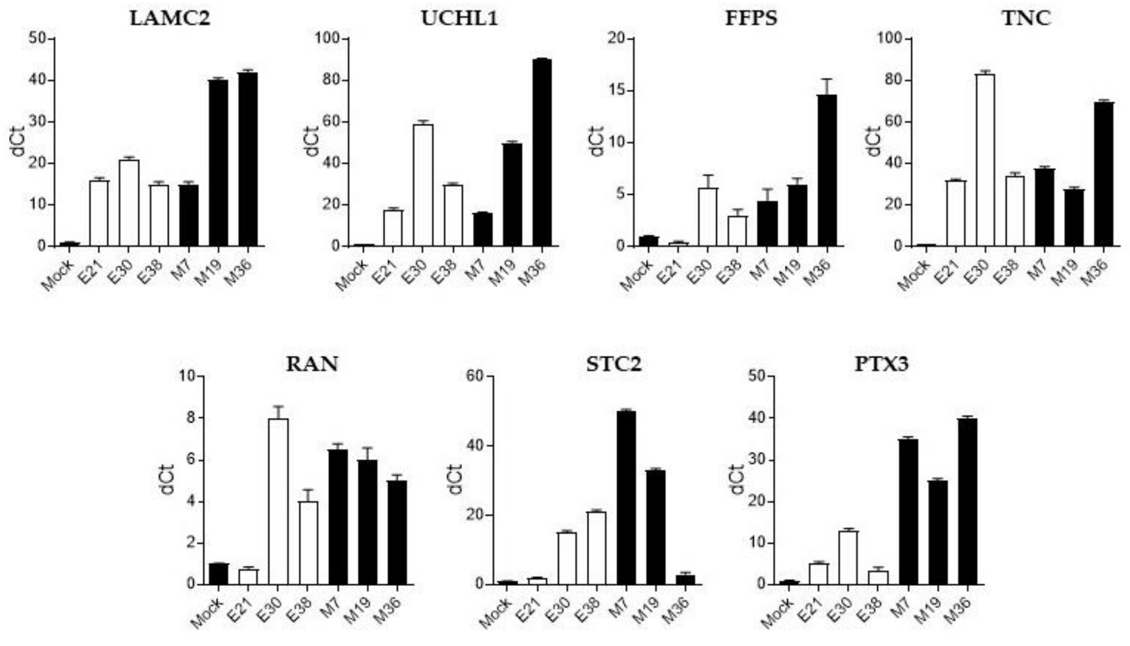

Figure S4. mRNA expression levels of the candidate biomarker in the KRAS-cloned lines

mRNA expression of the 7 selected proteins in different cloned HPDE-KRAS^{G12V} cell lines. White bars correspond to cloned lines with epithelial features; black bars to cloned lines with mesenchymal features (Ref. 41).

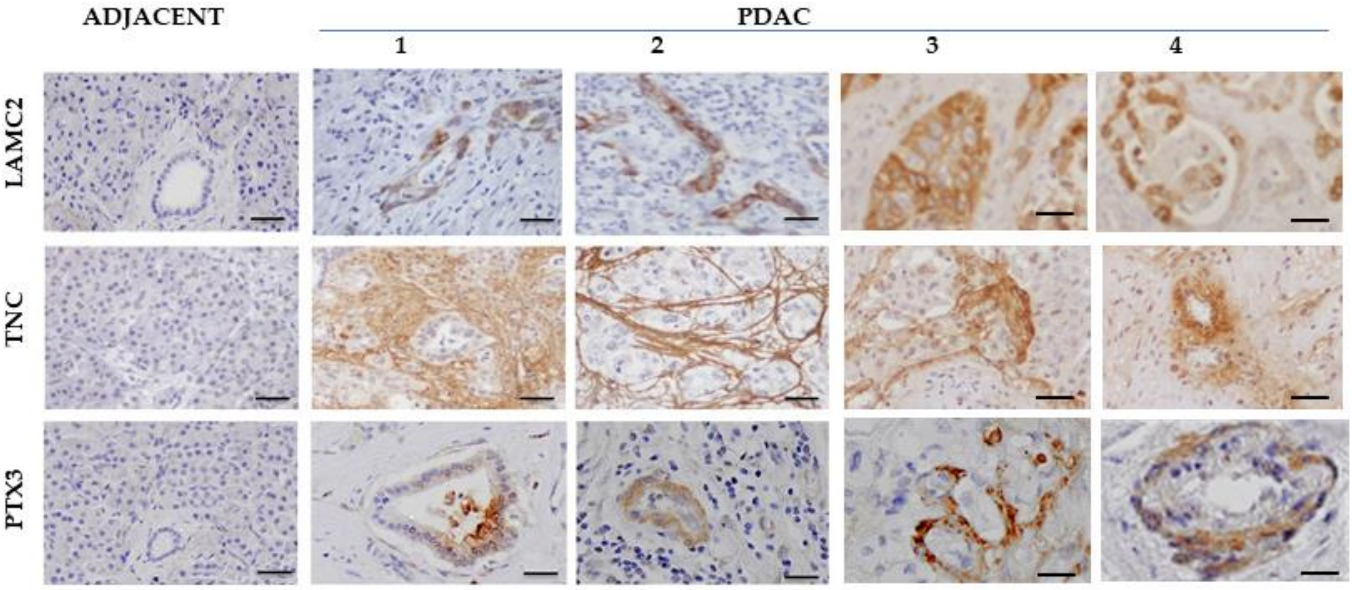

Figure S5. **Expression of LAMC2, TNC and PTX3 in PDAC tissues.**
Representative pictures of immunohistochemistry for LAMC2, TNC and PTX3 in PDAC surgical samples and adjacent pancreatic tissues showing positive immunostaining in tumor cells and for TNC also in the stroma.

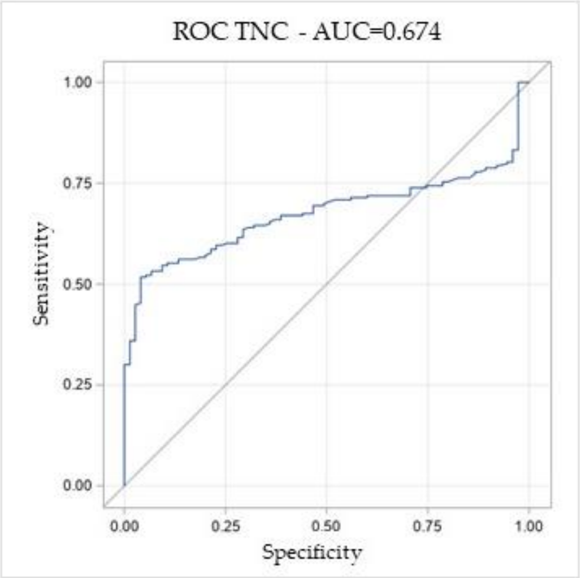

Figure S6. Sensitivity and specificity of the candidate biomarker TNC,
Sensitivity and specificity of TNC analyzed by the Area Under Curve (AUC) of receiver operating characteristics (ROC) curves. Analyses include PDAC patients (n=200) and healthy individuals (n=75).

References

1-Schiarea S, Solinas G, Allavena P, Scigliuolo GM, Bagnati R, Fanelli R, Chiabrando C. Secretome analysis of multiple pancreatic cancer cell lines reveals perturbations of key functional networks. J Proteome Res. 2010 Sep 3;9(9):4376-92. doi: 10.1021/pr1001109. PMID: 20687567
