# Supplementary figures and images for: Oncogenic KRAS-Induced Protein Signature in the Tumor Secretome Identifies Laminin-C2 and Pentraxin-3 as Useful Biomarkers for the Early Diagnosis of Pancreatic Cancer
Source: Cancers (Basel). 2022 May 27;14(11):2653. doi: 10.3390/cancers14112653 (PMC9179463; doi:10.3390/cancers14112653)

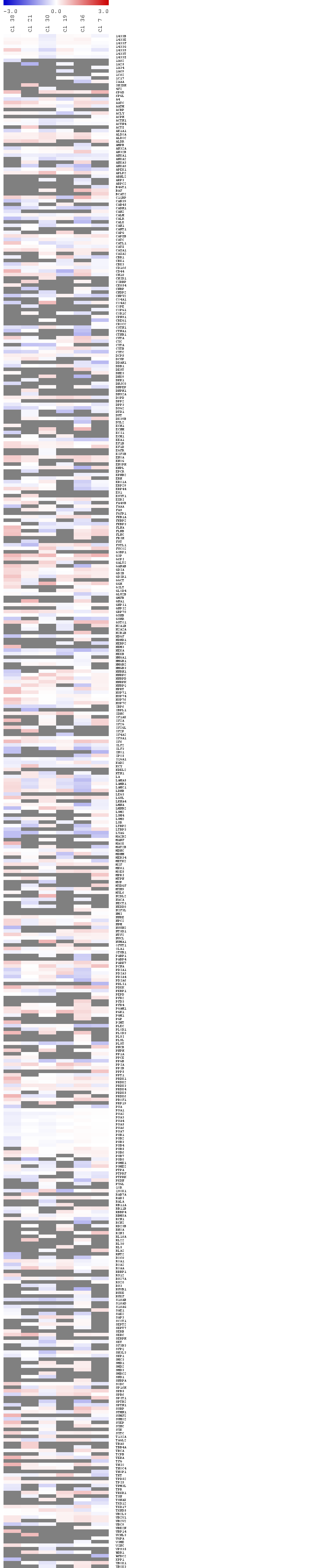

Supplement: Supplementary file 1 [file cancers-14-02653-s001.zip › Heatmap S1 437 non-dysregulated proteins in KRAS cloned lines.png]

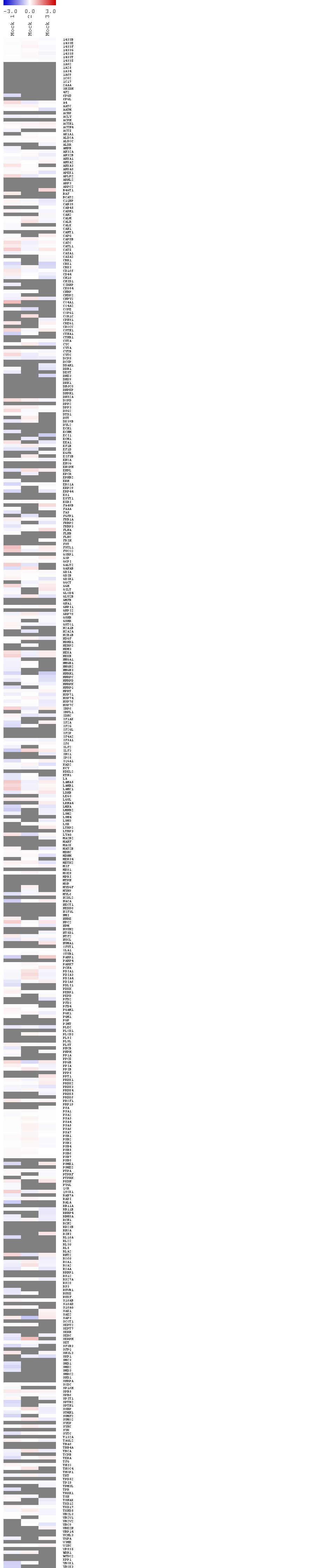

Supplement: Supplementary file 1 [file cancers-14-02653-s001.zip › Heatmap S2 437 non-dysregulated proteins in Mock lines.png]

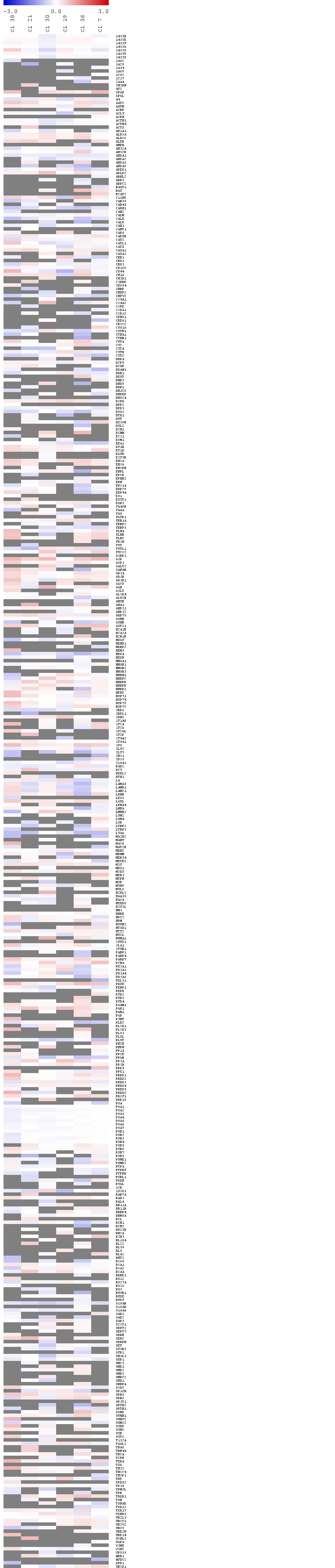

Supplement: Supplementary file 1 [file cancers-14-02653-s001.zip › Heatmap S3 Global 600 identified proteins in KRAS cloned lines.png]

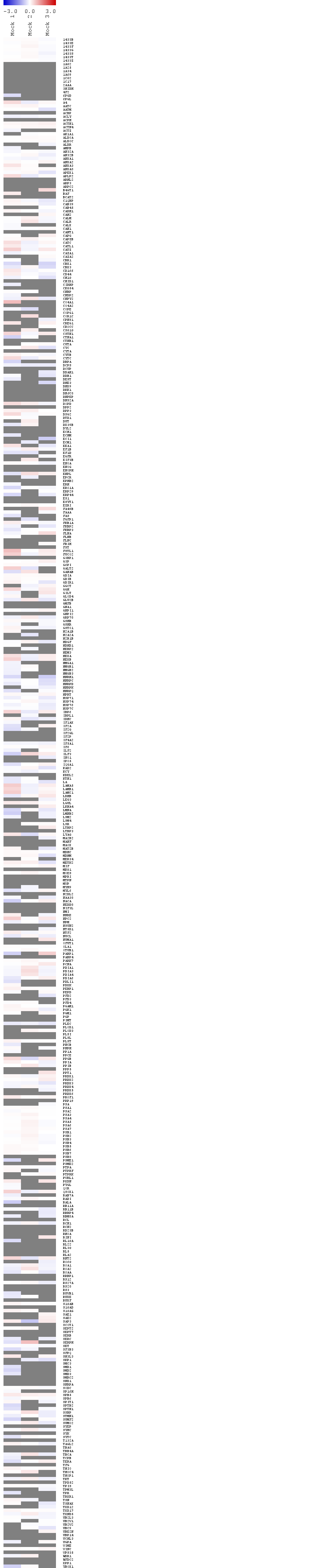

Supplement: Supplementary file 1 [file cancers-14-02653-s001.zip › Heatmap S4 Global 600 identified proteins in Mock lines.png]
